# Frailty as a Predictor of Outcomes in Optiflow

**DOI:** 10.7759/cureus.95828

**Published:** 2025-10-31

**Authors:** Matthew Mort, Alwin Raju, Naina Mohan

**Affiliations:** 1 Cardiology, Kingston Hospital, London, GBR; 2 Acute Internal Medicine, Kingston Hospital, London, GBR

**Keywords:** acute hypoxemic respiratory failure, care of the elderly, clinical frailty, high-flow nasal cannula (hfnc), mortality, pneumonia

## Abstract

High-flow nasal cannula (HFNC) oxygen therapy has been increasingly used in acute settings for patients with hypoxic respiratory failure. More evidence is needed to identify which patients are more likely to benefit from HFNC over conventional oxygen therapy. There is limited published evidence regarding the impact of frailty on outcomes in patients treated with HFNC. Does a higher Clinical Frailty Score confer a worse outcome in those treated with HFNC?

A retrospective cohort study of 96 patients who received HFNC was conducted at Kingston Hospital. Patients were divided into three groups based on their Clinical Frailty Score (CFS): CFS 1-4 (n=32), CFS 5-6 (n=44), and CFS 7-8 (n=20). Chi-squared test of independence was performed for significance in categorical variables, and for continuous variables, non-parametric testing using the Kruskal-Wallis H test was performed.

The 30-day mortality in the CFS 7-8 group was found to be higher (55%) as opposed to the CFS 1-4 group (41%) and CFS 5-6 (55%) (p=0.48). Frail patients with a pneumonic process had a higher 30-day mortality rate than non-frail patients (8 of 12 or 75% of patients with CFS 7-8 p=0.034) and when compared to frail patients with non-pneumonic processes (2 of 7 or 29% of patients with CFS 7-8 p=0.18). Mortality in the hospital whilst receiving HFNC was higher in the frailer groups (CFS 5-6: 18 of 44 (41%); CFS 7-8: 6 of 20 (30%)) compared to the less frail group (CFS 1-4: 6 of 32 (19%)) (p=0.360).

Our results suggest that frail patients experienced a higher mortality rate and lower weaning rate, particularly if the indication for HFNC was an underlying pneumonic process, suggesting that while HFNC provides benefits, its efficacy may be limited. Further evidence is needed to assess the efficacy of HFNC in frailty.

## Introduction

The use of high-flow nasal cannula for hypoxaemic respiratory failure has been on the rise recently. Systems of delivery such as Optiflow (Fisher & Paykel, Auckland, New Zealand) have become more widely available, allowing for more liberal use in the acutely hypoxic patient. Initially, during the 1990s, a growing body of evidence showed that non-invasive ventilation (NIV) proved superior outcomes when treating chronic obstructive pulmonary disease exacerbations, acute cardiogenic pulmonary oedema and immunocompromised respiratory failure compared with conventional oxygen supplementation. In the 2000s, an alternative oxygen delivery method was developed, allowing for greater flow rates via high-flow nasal cannula (HFNC) [1]. Given its novelty, the knowledge base is still expanding. The benefit of HFNC has been well established over conventional oxygen therapy in acute hypoxaemic respiratory failure (AHRF) via several systematic reviews and meta-analyses. HFNC has been shown to improve oxygenation, patient comfort and work of breathing [2,3]. It can also be used as a bridge prior to initiating NIV. It also conveys a decreased risk of intubation or escalation of oxygen therapy, although it does not reduce length of stay (including in the intensive care unit, high dependency unit, and medical wards) [4].

The most widely used method of HFNC oxygen delivery in the NHS is Optiflow, a system capable of delivering heated, humidified air and oxygen at flow rates up to 70L/min. To determine indications for Optiflow as a primary support in medical patients, numerous guidelines have been proposed [5-12]. These demonstrate Optiflow's benefit as a pre- and de-escalation support from invasive intubation. They also suggest benefit in, and propose guidelines for patients with a number of conditions including acute respiratory failure (generic recommendation for patients who require oxygen) [5], patients who are immunocompromised with AHRF (generic recommendation for NHF or COT) [5], acute respiratory distress syndrome (ARDS) [12], acute hypoxaemic respiratory failure [6], [7,10,11], acute hypercapnic respiratory failure [7], sepsis-induced AHRF [8] and patients with severe or critical COVID-19 [9].

Despite the clear benefits of HFNC, more evidence is needed to identify which patient cohorts are likely to benefit from HFNC over conventional oxygen therapy, as acknowledged by Rochwerg et al. [6]. There seems to be limited information as to which studies included exclusion criteria for frail patients or discussed discrepancies in patient outcomes based on frailty.

The UK has an ageing population, and patients with chronic conditions are living longer, resulting in a greater proportion of patients being admitted to hospital with a higher degree of frailty. Having a greater degree of frailty is also associated with worse outcomes for hospitalised patients; they stay longer and have a higher mortality rate [13]. It has become good practice to consider frailty scores for patients admitted to the hospital to aid in treatment escalation plans (TEP), and the Rockwood Frailty Scale has become the gold standard [14].

At our centre (Kingston Hospital), several TEPs suggesting "trial of Optiflow" as a ceiling of care have been noted, and several patients reviewed by the Critical Care Outreach Team deemed too frail for intubation and the ICU have died with Optiflow still in use. It is important to note the discrepancy between age and frailty, as less frail, more elderly ARF patients may still benefit from HFNC [15]. Fimognari et al.'s case series of 18 patients determined that HFNC did not improve outcomes in cases of extreme premorbid disability. In fact, there is some suggestion that HFNC may have been associated with harm for elderly, frail patients with severe COVID-19 pneumonitis, inappropriate for invasive ventilation [16].

This study was conducted to add to the growing body of evidence investigating whether frailty is a negative predictor of outcomes in Optiflow; we aim to determine whether patient frailty has a bearing on the mortality of patients receiving Optiflow (with 30-day mortality as a primary outcome). Our data will also be used to compare frailty and secondary outcomes, including duration of treatment, outcome of treatment and patient location at 30 days. 

## Materials and methods

A retrospective, cohort study was conducted at Kingston Hospital of 96 patients requiring Optiflow in a 12-month period between January and December 2023. Patients commenced on Optiflow in intensive care were excluded. Optiflow is frequently used as a bridge post-extubation and not solely as a treatment for acute respiratory failure in intensive care. Furthermore, admission criteria to the ICU will also take into consideration a patient's frailty; this may therefore create a bias in the proportion of frail vs non-frail patients receiving Optiflow, and their subsequent morbidity and mortality. All patient notes at Kingston Hospital are electronic, allowing for retrospective auditing via an individualised patient record number; the patient record numbers of all patients initiated on Optiflow were recorded in the 12-month period by the Critical Care Outreach Team. Prior to initiation of Optiflow, an electronic pro forma is completed outlining the indication for treatment and stored within the electronic record. As a matter of good practice, the Clinical Frailty Score is recorded at the time of admission clerking.

Patients were divided into three groups based on their Clinical Frailty Score (CFS). A patient's frailty was either formally documented as part of the admission clerking or determined from their social history and inpatient review and allocated as per Rockwood's Clinical Frailty Score [15]. Within each CFS group, the specific indication for Optiflow was recorded (either from the initiation pro-forma or from ward round entries) along with the duration of HFNC treatment. All anonymised data was collected and collated via a shared spreadsheet by the authors and contributors. 

The primary outcome compared between CFS groups was 30-day mortality; secondary outcomes were defined as duration on Optiflow (days), outcome whilst on Optiflow (weaned, died, intubated, did not tolerate) and location at 30 days (inpatient vs outpatient vs deceased). "Weaned" was defined as treatment cessation due to resolution of respiratory failure and considered the final day that Optiflow therapy was received (regardless of any intermitted breaks in therapy prior to this). A comparison of 30-day mortality was also made between the community-acquired pneumonia (CAP)/aspiration/hospital-acquired pneumonia (HAP) and non-CAP indications for Optiflow, i.e., "pneumonic" and "non-pneumonic" processes. 

All data were analysed using descriptive and inferential statistics. Continuous variables, such as patient age and duration of high-flow nasal oxygen (HFNO) therapy, were summarised as means with standard deviations (SD) if normally distributed, or as medians with interquartile ranges (IQR) when distributions were skewed. Categorical variables, including sex, indication for HFNO, in-hospital outcome, 30-day mortality and location at 30 days, were presented as absolute numbers with corresponding percentages.

Comparisons between frailty groups were based on CFS subsets. For categorical variables, associations across frailty groups were tested using the Chi-squared (χ²) test of independence. The chi-square statistic and the corresponding p-value are reported within each table in Results. For continuous variables, non-parametric testing was performed using the Kruskal-Wallis H test, given the small sample sizes and potential non-normal distributions. Results are reported as test statistics (χ² or H values) together with p-values.

A p-value of <0.05 was considered statistically significant, while results with p<0.001 were regarded as highly significant. Where no valid test or data point was applicable to individual cell counts, results are indicated with a dash (-) in the tables.

As this was a retrospective notes review, ethical approval was not required at our institution. Potential confounders within the data include age, co-morbidities and functional baseline; however, the Clinical Frailty Score collates these variables when clinicians are determining a patient's score. As the Clinical Frailty Score is a validated score consisting of a number of variables, it was considered a strong exposure variable for the Chi-squared and Kruskal-Wallis tests. 

## Results

As Table 1 demonstrates, the mean age across the three CFS groups was 79, 82 and 81 years, respectively. The most common indications for HFNC were CAP/HAP; this constituted 17 (53%), 20 (45%) and eight (40%) of the patients in each CFS group, respectively.

**Table 1:** Baseline characteristics by frailty group (CFS) p-values are based on Chi-squared tests for categorical data and Kruskal–Wallis tests for continuous data. Significance thresholds: p<0.05 (*), p<0.001 (**) CAP - community-acquired pneumonia; HAP - hospital-acquired pneumonia; HFNO - high-flow nasal oxygen; CFS - Clinical Frailty Score

Characteristic	CFS 1–4 (n = 32)	CFS 5–6 (n = 44)	CFS 7–8 (n = 20)	χ² / H	p-value
Age in years, mean ± SD	78.6 ± 8.1	82.3 ± 7.0	81.1 ± 6.1	H=5.85	0.054
Sex (F/M)	12 / 20	21 / 23	7 / 13	3.62	0.728
Primary indication, n (%)	-	-	-	12.58	0.400
CAP/HAP	17 (53%)	20 (45%)	8 (40%)	-	-
COVID pneumonitis	3 (9%)	4 (9%)	5 (25%)	-	-
Aspiration	2 (6%)	12 (27%)	4 (20%)	-	-
Pulmonary oedema	6 (19%)	5 (11%)	2 (10%)	-	-
Other (fibrosis/PE/not doc)	4 (13%)	3 (7%)	1 (5%)	-	-
Duration of HFNO in days, median (IQR)	3.0 (2-6)	2.0 (1-5)	2.0 (1-4)	H=2.56	0.278

Table 2 compares primary and secondary outcomes based on Clinical Frailty Score and shows the 30-day mortality in the CFS 7-8 group was found to be higher (11 of 20 patients or 55% deceased at 30 days) when compared to the less frail CFS 1-4 group (13 of 32 patients or 41% deceased at 30 days) (p=0.482). The less frail patients also had better outcomes; 22 of 32 patients, or 69% in CFS 1-4, were weaned off HFNC, compared to 22 of 44 (50%) and 12 of 20 (60%) in groups CFS 5-6 and CFS 7-8, respectively. Mortality whilst receiving Optiflow in hospital within the frailer groups was higher (18 of 44 or 41% in CFS 5-6 and 6 of 20 or 40% in CFS 7-8) than that of the less frail group (6 of 32 or 19% in CFS 1-4) (p=0.360). HFNC was well tolerated by all patients, regardless of frailty.

**Table 2:** Outcomes by frailty group (CFS) p-values are based on Chi-squared tests for categorical data and Kruskal–Wallis tests for continuous data. Significance thresholds: p<0.05 (*), p<0.001 (**) CFS - Clinical Frailty Score

Outcome	CFS 1-4 (n=32)	CFS 5-6 (n=44)	CFS 7-8 (n=20)	χ²	p-value
30-day mortality, n (%)	13 (41%)	24 (55%)	11 (55%)	11.55	0.482
In hospital outcome, n (%)	-	-	-	8.79	0.360
Weaned	22 (69%)	22 (50%)	12 (60%)	-	-
Died	6 (19%)	18 (41%)	6 (30%)	-	-
Intubated	3 (9%)	3 (7%)	1 (5%)	-	-
Did not tolerate	1 (3%)	1 (2%)	0	-	-
30-day location, n (%)	-	-	-	2.86	0.582
Home	16 (50%)	14 (32%)	7 (35%)	-	-
Inpatient	3 (9%)	6 (14%)	2 (10%)	-	-
Deceased	13 (41%)	24 (55%)	11 (55%)	-	-

The duration of high-flow nasal oxygen therapy differed significantly across Clinical Frailty Score (CFS) groups (Kruskal-Wallis H=2.56, p=0.278). Patients with lower frailty scores had longer median durations on HFNO compared to those with higher frailty scores.

A further subgroup analysis was performed comparing 30-day mortality in the context of pneumonic processes (CAP/HAP/aspiration) requiring Optiflow, as outlined in Table 3. Within the context of a pneumonic process, the CFS 1-4 group had a 30-day mortality of six out of 19 deceased at 30 days (32%), the CFS 5-6 group had 15 of 32 (45%), and the CFS 7-8 group had eight of 12 (75%) (p=0.034).

**Table 3:** 30-day mortality in patients with pneumonic and non-pneumonic indications p-values are based on Chi-squared tests for categorical data and Kruskal–Wallis tests for continuous data. Significance thresholds: p<0.05 (*), p<0.001 (**) CFS - Clinical Frailty Score

	CFS	1-4	5-6	7-8	All	p-value
30-day mortality, n (%)	Pneumonic	6 (32%)	15 (45%)	8 (75%)	29 (46%)	0.034*
	Non-pneumonic	7 (54%)	7 (58%)	2 (29%)	9 (50%)	0.18

## Discussion

Our results suggest HFNC is an effective and well-tolerated intervention, but outcomes are influenced by the frailty of patients. The higher successful wean and discharge rates in less frail patients (CFS 1-4) indicate a better prognosis compared to more frail patients (CFS 5-6 and 7-8). Frail patients (CFS 5-8) demonstrated a higher mortality rate and lower weaning rate, suggesting that while HFNC provides benefits, its efficacy may be limited in more frail individuals.

The study is limited as a single centre, retrospective, non-randomised review. As such, the statistical significance of our results has been affected. When patients were stratified into the three frailty categories, no statistically significant differences were observed in the majority of clinical outcomes, including 30-day mortality. Although patients with higher frailty scores tended to have a higher 30-day mortality, these differences did not reach statistical significance. These findings suggest that, within this cohort, frailty as measured by the Clinical Frailty Scale was not independently associated with differences in HFNO treatment outcomes. However, when focusing on pneumonic processes (community-acquired pneumonia, hospital-acquired pneumonia, and aspiration pneumonia), a statistically significant difference was observed in 30-day mortality for patients receiving Optiflow, with frailty conveying a worse outcome. 

Some clinicians will not offer HFNC to frail patients as part of their current practice, and this will have affected our results. Further evidence is needed to assess the efficacy of HFNC in frailty. Given the deviation of three years between the mean ages of each frailty group (CFS 1-4: 79 years, CFS 5-6: 82 years, and CSF 7-8: 81 years), we can deduce that age alone is not a suitable predictor of outcomes, despite it often being considered a potential confounder. There is also a limited number (19) of more frail patients (CFS 7-8) receiving Optiflow, potentially limiting the study's power. Further studies across multiple centres could improve this. While our study does suggest trends in mortality for more frail patients in non-pneumonic processes, we can more definitively suggest a correlation in pneumonic processes and frailty; a larger cohort of patients will add power to these suggested trends.

Further confounders within the data include co-morbidities and functional baseline; however, the Clinical Frailty Score is a collation of these variables. As the CFS is based on a clinician's review of these variables, there is inherent subjectivity in determining a patient's score. Despite this, as the Clinical Frailty Score is a validated score consisting of these variables, it was considered to be a strong exposure variable for Chi-squared and Kruskal-Wallis tests. A multiple clinician scoring system with blinding could reduce any bias or subjectivity within score allocation. Severity of disease and body mass index (BMI) may also confound results, but there was insufficient data within patient records to accurately include these for multivariable analyses; a prospective study with this data included would help strengthen the relationship analysis between frailty and outcomes.

There is evidence available demonstrating HFNC's effectiveness in pneumonia and pneumonic processes, including the FLORALI trial [17] comparing HFNC to conventional low flow oxygen therapy and non-invasive ventilation (NIV). This study included ITU patients, but did not comment on frailty within the patient cohort studied. Frailty is an important predictor of outcomes; Yang et al. [18] have demonstrated via meta-analysis that frailty itself is associated with pneumonia and can also be a predictor of poor outcomes. Within their meta-analysis of 16 studies, 50% of older patients with pneumonia were found to be frail, and the frailer patient cohort had an odds ratio for mortality of 3.51. This high-risk, frail cohort of pneumonia patients also exhibited significantly longer durations of admission and higher chances of readmission. Therefore, while HFNC may be effective in undifferentiated pneumonia patients, a more detailed analysis of outcomes in frail patients with pneumonia is required.

Some work has been done in constructing prediction tools for HFNC in the context of specific conditions, including pneumonia with acute hypoxaemic respiratory failure [19], and much of the body of work thus far using frailty as a predictor is in the context of COVID-19 infection [20], [21]. Although small, single-centre studies, Bouetard et al. [20] allude to lower frailty resulting in improved outcomes (but acknowledge the ease of use in Optiflow even in frail patients), and van Steenkiste et al. [21] suggest HFNC as a rescue therapy for patients with COVID-19 infection not suitable for ICU. Furthermore, Type 1 respiratory failure to the extent of requiring Optiflow should be recognised as a sign of deterioration and perhaps initiate discussion regarding the ceiling of treatment, as opposed to a rescue therapy [22]. 

## Conclusions

One can infer that a Clinical Frailty Score should be considered when determining treatment escalation plans for patients on admission and when determining how appropriate patients may be for HFNC in the context of acute hypoxic respiratory failure. This casual relationship could also form the hypothesis for future, larger and prospective studies into the relationship between frailty and Optiflow outcomes.

Given our ageing population and the wider availability of Optiflow, there is a growing need to ensure this treatment conveys benefit to frail patients. Optiflow use should be considered as part of a patient's treatment escalation plan; a requirement for HFNC indicates a patient is deteriorating, and while its use may provide benefit, there should also be a recognition of the potential for mortality. If a clear correlation between frailty and mortality can be established in Optiflow patients, further tools such as decision aids could be developed to help guide clinicians when treating the acutely deteriorating patient.
